# *In Vivo* Antioxidant Activity of Deacetylasperulosidic Acid in Noni

**DOI:** 10.1155/2013/804504

**Published:** 2013-11-24

**Authors:** De-Lu Ma, Mai Chen, Chen X. Su, Brett J. West

**Affiliations:** ^1^Division of Pharmacology, Tianjin Medical University, Tianjin 300070, China; ^2^Quality Control, Tahitian Noni Beverages Company Ltd., Room A 12F, No. 789, Zhaojiabang Road, Shanghai 200032, China; ^3^Research and Development, Morinda Inc., 737 East 1180 South, American Fork, UT 84003, USA

## Abstract

Deacetylasperulosidic acid (DAA) is a major phytochemical constituent of *Morinda citrifolia* (noni) fruit. Noni juice has demonstrated antioxidant activity *in vivo* and in human trials. To evaluate the role of DAA in this antioxidant activity, Wistar rats were fed 0 (control group), 15, 30, or 60 mg/kg body weight per day for 7 days. Afterwards, serum malondialdehyde concentration and superoxide dismutase and glutathione peroxidase activities were measured and compared among groups. A dose-dependent reduction in malondialdehyde was evident as well as a dose-dependent increase in superoxide dismutase activity. DAA ingestion did not influence serum glutathione peroxidase activity. These results suggest that DAA contributes to the antioxidant activity of noni juice by increasing superoxide dismutase activity. The fact that malondialdehyde concentrations declined with increased DAA dose, despite the lack of glutathione peroxidase-inducing activity, suggests that DAA may also increase catalase activity. It has been previously reported that noni juice increases catalase activity *in vivo* but additional research is required to confirm the effect of DAA on catalase. Even so, the current findings do explain a possible mechanism of action for the antioxidant properties of noni juice that have been observed in human clinical trials.

## 1. Introduction

*Morinda citrifolia*, commonly known as noni, is a small tree that has been used as a traditional source of food and medicine throughout the tropics [1, 2]. A variety of potential health benefits have been reported for noni fruit juice [3]. These include immunomodulation [4, 5] and antioxidant activities *in vitro* and *in vivo* [6–8]. The antioxidant activity of noni juice was found to be associated with increased endurance in athletes [9]. In a human clinical trial involving heavy cigarette smokers, consumption of noni juice resulted in lowered plasma concentrations of superoxide anion radicals (SAR) and lipid hydroperoxides [10]. Further, consumption of noni juice also decreased the level of lipid peroxidation-derived DNA adducts in the lymphocytes of heavy smokers [11].

*In vivo* research has demonstrated that noni juice increases superoxide dismutase (SOD) and glutathione peroxidase (GPx) enzyme activities [12]. The superoxide anion radical (SAR) is a major cellular reactive oxygen species and may be generated via enzymatic and nonenzymatic process or may come from exogenous sources, including cigarette smoke [13]. SOD catalyzes the dismutation of SAR to hydrogen peroxide and oxygen [14]. GPx is capable of reducing free hydrogen peroxide to water [15]. GPx also reduces lipid hydroperoxides, as well as prevents free radical attack on polyunsaturated fatty acids in cellular membranes [16]. As such, the effect of noni juice on these two enzymes may be at least two of the major antioxidant mechanisms of action through which it protects lymphocyte DNA and lowers plasma concentration of tobacco smoke-induced free radicals and peroxides.

Chemical studies of noni fruit have revealed that iridoids are the main phytochemical constituents, with deacetylasperulosidic acid (DAA) comprising the majority of the iridoid content [17]. DAA has anticlastogenic activity, suppressing the induction of chromosome aberrations in Chinese hamster ovary cells and in mice [18]. DAA is reported to inhibit the release of tumor necrosis factor-alpha from cultured mouse peritoneal macrophages and inhibits low-density lipoprotein oxidation [19, 20]. DAA also prevented 4-nitroquinoline 1-oxide (4NQO) induced DNA damage *in vitro* [21]. 4NQO exposure leads to the formation of superoxide, hydrogen peroxide, and hydroxyl radicals, resulting in the production of a substantial amount of 8-hydroxydeoxyguanosine, a product of DNA oxidation in mammalian and bacterial cells [22, 23]. Treatment with DAA reduced 4NQO genotoxicity by 98.96%, suggesting that iridoids are responsible for the DNA protective effects of noni juice in cigarette smokers. 

With demonstrated antioxidant activity of noni juice and the potential bioactivities of its major phytochemical constituent, the current study was conducted to investigate the role of DAA on SOD and GPx activities *in vivo*.

## 2. Materials and Methods

### 2.1. Test Material

Deacetylasperulosidic acid (DAA) was obtained from Chengdu Biopurify Phytochemicals Ltd. (Chengdu, China). The purity of DAA was 98%, and the identity was confirmed by high performance liquid chromatography [24]. DAA was dissolved in MeOH-H_2_O (1 : 1) at a concentration of 0.2 mg/mL. Separation of the standard was performed with a HC-C18 column (25 cm × 4.6 mm; 5 *μ*m, Agilent Technologies, Santa Clara, CA, USA) in a Waters 2690 separations module (Waters Corporation, Milford, MA, USA) and detected with a Waters 2489 UV/Vis detector at 235 nm. DAA was eluted at a flow rate of 0.8 mL/min with two mobile phases: (A) MeCN and (B) 0.1% formic acid in H_2_O (v/v). The elution gradient was 5 min with 100% B, 35 min with 30% A and 70% B, 12 min 95% A and 5% B, and then 8 minutes with 100% B. The retention time and absorbance spectrum were compared against those of a DAA standard.

### 2.2. Animals and Treatment

For this study, 40 Wistar rats (male and female, 180–200 g) were obtained from the Experimental Animal Center, Academy of Military Medical Sciences (Beijing), Approval no. SCXK-Army-2009-003. The care of animals and experimental procedures were compliant with ethical and institutional animal welfare guidelines of Tianjin Medical University. Following acclimation, rats were randomly divided into 4 groups of 10 each (5 male and 5 female). Each group was provided feed and water *ad libitum*. DAA was dissolved in saline and each animal was gavaged for 7 days with 1 mL/200 g body weight (bw) of one of four treatments, depending on group assignment. The treatments were normal saline (control), 15 mg DAA/kg bw (low dose), 30 mg DAA/kg bw (mid dose), and 60 mg DAA/kg bw (high dose). Individual animal weight and feed intake were recorded on days 1, 4, and 7. On the 8th day, the rats were anesthetized and 0.5 mL blood was removed from the orbital sinus. Whole blood was centrifuged at 3,000 rpm for 10 min in a low speed centrifuge (model BFX5-320, Baiyang Centrifuge Factory, Liulizhuang, Hebei, China). The resulting serum was retained for assays.

### 2.3. Antioxidant Enzyme Activity Assays and Malondialdehyde Assay

Using a commercial bioassay kit (Jiancheng Bioengineering Institute, Nanjing, China), the SOD activity of serum was assayed. The superoxide dismutase activity assay utilized the production of a water soluble dye (WST-1 formazan) from a tetrazolium salt (WST-1) in the presence of SAR [25]. In a phosphate-buffered reaction mixture, xanthine and xanthine oxidase are incubated at 37°C in the presence of samples, blanks, SOD standards, and WST-1. During the reaction, SAR is generated from xanthine and oxygen. SOD catalyzes the dismutation of SAR, thereby reducing the amount available to oxidize WST-1 to WST-1 formazan. Sample results were compared against an SOD standard reference curve after the absorbance was read at 450 nm with a microplate reader (Epoch Microplate Spectrophotometer, BioTek, Winooski, VT, USA). The SOD activity is determined by calculating the WST-1 formazan inhibition rate and is expressed in U/mL. 

GPx activities in serum samples were also determined with a commercially available bioassay kit (Jiancheng Bioengineering Institute, Nanjing, China). GPx activity was measured by determining reduced glutathione in the serum. Absorbance of sample, blanks, and standards were read at 412 nm with a microplate reader (Epoch Microplate Spectrophotometer, BioTek, Winooski, VT, USA). Enzyme activities were expressed as U/mL.

Malondialdehyde (MDA) concentrations were assayed with a commercial bioassay kit (Jiancheng Bioengineering Institute, Nanjing, China). MDA was detected according to the common colorimetric method involving the reaction of samples and 1,3,3,3 tetra-ethoxypropane standards with thiobarbituric acid in a 95°C water bath for 40 min. The absorbance of each of the samples and standards was measured at 532 nm (Epoch Microplate Spectrophotometer, BioTek, Winooski, VT, USA). Results were determined against the standard curve and expressed as nmol/mL. 

### 2.4. Statistical Analysis

Basic summary statistics (mean, range, and standard deviation) were calculated. Intergroup differences were measured with Student's *t*-test, following Bartlett's test for homogeneity of variance. 

## 3. Results and Discussion

Weights and feed intake rates among the different groups are compared in Tables 1 and 2. All groups experienced appropriate weight gain during the seven-day period [26], with no differences between any groups. There were no intragroup changes in mean feed intake. There were no feed intake differences between the groups on days 1 and 4. The low and mid dose groups had slightly lower feed intakes on day 7 than the control group, but these were within the expected amounts for healthy Wistar rats [27]. There was no significant difference in feed intake between the control and high dose groups.

The effects of DAA intake on MDA concentration and antioxidant enzyme activities are summarized in Table 3. While mean serum MDA content in the low dose group is lower than that of the control group, the difference is not statistically significant. However, there is a trend of reduced serum MDA concentrations with increased DAA dose. Serum MDA of the mid and high dose groups was significantly lower than those observed in the controls. The approximate percent reductions in mean serum MDA in the mid and high dose groups were, respectively, 19.7 and 22.6% when compared to the control group.

Mean serum SOD activity tended to increase with DAA dose. While SOD activity in low dose group was almost 10% greater than that in the controls, the difference was only marginally significant (*P* = 0.098). On the other hand, SOD activities observed in the mid and high dose groups were significantly greater than that of the control group. Mid and high dose animals experienced, respectively, an average increase of 11.3 (*P* < 0.05) and 13.0% (*P* < 0.01) in serum SOD activity. Unlike SOD, GPx activities were not influenced by DAA intake at any of the doses evaluated.

DAA has apparent antioxidant activity when ingested. The effect of DAA on SOD activity helps to explain at least one mechanism whereby noni juice lowered plasma SAR in heavy smokers. SAR contributes to the eventual formation of MDA by means of reactive hydroxyl radicals formed either through the reduction of transition metal ions by SAR or from the degradation of peroxynitrite that is produced in reactions between SAR and nitric oxide [28]. Therefore, the increased dismutation of SAR to H_2_O_2_ and oxygen will lower the amount available for the formation of MDA. This suggests that one way in which DAA decreases lipid peroxidation, as measured by lower MDA levels, is by increasing SOD activity. This is further evidenced by the observed DNA protective effect of noni juice in heavy smokers, as MDA reacts readily with DNA bases to form lipid peroxidation-derived DNA adducts [11, 29].

One very interesting observation of this study is the inability of DAA to increase GPx activity. Increased SOD activity results in a decrease in SAR concentrations. But a consequence of SOD activity is the increased H_2_O_2_. H_2_O_2_, in turn, can cause oxidative damage, if not further metabolized into less reactive compounds. Elevated H_2_O_2_ has been found to increase MDA concentrations via lipid peroxidation [30]. But MDA decreased in our study, even though GPx activity was unaffected. Therefore, another pathway must have been involved in the control of H_2_O_2_ levels. Catalase is another antioxidant enzyme which promotes the degradation of H_2_O_2_ into water and oxygen [31]. Therefore, it may be that catalase activity is increased by DAA. This possibility is further supported by the fact that *Morinda citrifolia* leaves contain DAA [17] and that catalase activity was increased in lymphoma-bearing mice which had been fed crude *M. citrifolia* leaf extract [32]. Additionally, an aqueous extract of *M. citrifolia* leaves increased catalase activity in hyperlipidemic rats [33]. Other iridoids have also been reported to increase catalase activity [34], so this is a bioactivity associated with this class of compounds. 

The results of this experiment demonstrate that DAA exerts an antioxidant effect by increasing SOD activity. This effect may be responsible, at least in part, for the antioxidant properties of noni juice as demonstrated in human trials and *in vivo*. The *in vitro* antioxidant activity of DAA against 4NQO may also be mediated through SOD activation, as the microorganism involved expresses this enzyme [35]. However, DAA does not appear to increase GPx activity by itself, at least under the conditions of this study. As discussed previously, noni juice increases GPx activity *in vivo*. Even though DAA is a major constituent of noni juice, the fruit also contains several other iridoids in minor concentrations [17, 36, 37]. Loganin, an iridoid similar in structure to epi-dihydrocornin found in noni fruit, increased GPx expression in rat mesangial cells that had been exposed to advanced glycation end products [38]. So, it is likely that another iridoid compound in noni, aside from DAA, is responsible for the increased GPx activity. It is also possible that interaction between several phytochemical constituents results in increased serum GPx. 

## 4. Conclusion

The *in vivo* antioxidant activity of DAA has been demonstrated via oral administration to Wistar rats for 7 days. A dose-dependent trend was evident, with significant reduction in serum MDA and an accompanying increase in SOD activity at 30 and 60 mg/kg bw. DAA did not, however, increase serum GPx activity. Since serum MDA declined with increased dose, it is possible that DAA induces catalase activity. However, additional research is required to confirm this. Animal weights and feed intake, along with previous toxicity tests of DAA, do not provide any indication of toxicity. The results of the current study suggest that DAA is responsible, at least in part, for the antioxidant activities observed in human trials. While it is possible that DAA's influence on SOD activity could explain the effect of noni juice on heavy smokers, the results also demonstrate that the effect of noni juice on GPx activity *in vivo* is not associated with DAA alone. Induction of GPx is likely due to another iridoid or a combination of phytochemical constituents. 

## Figures and Tables

**Table 1:** Mean (± standard deviation) weight (g) per animal.

DAA dose (mg/kg)	Day 1	Day 4	Day 7
0	189 ± 10.4	208 ± 21.2	220 ± 27.5
15	189 ± 8.26	207 ± 15.7	218 ± 22.2
30	189 ± 11.4	207 ± 20.7	217 ± 24.8
60	190 ± 8.97	207 ± 17.5	218 ± 24.0

**Table 2:** Mean (± standard deviation) feed intake (g) per animal.

DAA dose (mg/kg)	Day 1	Day 4	Day 7
0	21.9 ± 5.49	20.6 ± 5.62	21.8 ± 4.18
15	19.1 ± 3.49	19.8 ± 2.64	18.3 ± 2.65*
30	21.8 ± 5.86	21.6 ± 5.12	18.3 ± 2.41*
60	20.0 ± 4.66	19.1 ± 2.27	18.7 ± 2.40

*P < 0.05 compared to control group on day 7 but within expected feed intake rates.

**Table 3:** Mean (± standard deviation) serum MDA concentration and activity of antioxidant enzymes.

DAA dose (mg/kg)	MDA (nmol/mL)	SOD (U/mL)	GPx (U/mL)
0	5.54 ± 0.77	115 ± 10.1	995 ± 148
15	4.96 ± 1.43	126 ± 17.3	1007 ± 169
30	4.45 ± 1.15*	128 ± 15.2*	983 ± 167
60	4.43 ± 0.79**	130 ± 8.83**	986 ± 101

*P < 0.05,**P < 0.01 compared to control group.
